# Call combination order and iterations may shift meaning in sooty mangabey vocal sequences

**DOI:** 10.1186/s12915-026-02528-4

**Published:** 2026-02-21

**Authors:** Auriane Le Floch, Cédric Girard-Buttoz, Christof Neumann, Tanit Souha Azaiez, Natacha Bande, Roman M. Wittig, Klaus Zuberbühler, Catherine Crockford

**Affiliations:** 1 Ape Social Mind Lab, Institut Des Sciences, Cognitives Marc Jeannerod, CNRS, Université Lyon 1, Bron, France; 2 Institute of Biology, University of Neuchâtel, Neuchâtel, Switzerland; 3 Taï Chimpanzee Project, IRL 2041 ChiMP4IC CNRS, Centre Suisse de Recherches Scientifique en Côte d’Ivoire, Abidjan, Ivory Coast; 4 Taï Monkey Project, Centre Suisse de Recherches Scientifique en Côte d’Ivoire, Abidjan, Ivory Coast; 5 ENES Bioacoustics Research Laboratory, Centre de Recherche en Neurosciences de Lyon, UMR 5292, CNRS, Inserm, University of Saint-Etienne, Saint-Etienne, France; 6 Department of Human Behaviour, Ecology and Culture, Max Planck Institute for Evolutionary Anthropology, Leipzig, Germany; 7 Cognitive Ethology Laboratory, German Primate Center, 37077 Göttingen, Germany; 8 Department of Primate Cognition, Georg-August-Universität Göttingen, 37077 Göttingen, Germany; 9 Leibniz Science Campus Primate Cognition, 37077 Göttingen, Germany; 10 School of Psychology & Neuroscience, University of St Andrews, St Andrews, Scotland

**Keywords:** Compositionality, Language evolution, Vocal sequences, Meaning

## Abstract

**Background:**

Human language expands meaning through the structured combination of sounds, but such mechanisms remain rare in nonhuman animals, raising questions about their evolution. Shifts in meaning from single calls to combinations appear across a range of combinations and contexts in apes, but current evidence from other species is mainly restricted to alarm contexts. To address this, we applied a quantitative, whole-repertoire approach to assess meaningful combinatorial capacities in sooty mangabeys (*Cercocebus atys*), a forest-dwelling monkey.

**Results:**

We recorded 1751 vocal utterances from two groups in the Taï National Park, Ivory Coast. Using context of production as a proxy for potential meaning, we focused on two mechanisms: (1) bigrams—sequences of two different calls, and (2) iteration—reoccurrence of the same call type interspersed with others. Euclidean distance analyses suggested order-sensitive meaning in female bigrams combining ‘grunt’ and ‘twitter’ calls. Whereas single ‘grunts’, ‘twitters’ and the bigram ‘grunt_twitter’ occurred frequently during feeding, ‘twitter_grunt’ occurred predominantly during infant-directed affiliations. Female iterative sequences in which ‘grunt’ and ‘twitter’ calls recurred (e.g. ‘twitter_grunt_twitter’) also showed context-specific shifts, indicating a possible role for iteration in meaning generation.

**Conclusions:**

These findings suggest that meaningful combinatorial capacities can extend beyond great apes and alarm contexts to socially benign interactions in monkeys. However, repertoire-wide use of meaning-shifting bigrams remains unconfirmed outside hominids.

**Supplementary Information:**

The online version contains supplementary material available at 10.1186/s12915-026-02528-4.

## Background

Syntax, a key feature of language, allows humans to combine sounds into words and structured phrases, creating a potentially infinite range of meanings. The origins of this ability, unique to humans, remain difficult to reconstruct [1, 2]. Investigating whether rudimentary forms of syntax exist in non-human animals (hereafter animals) can provide insights into the evolutionary roots of our combinatorial communicative system [3–5].

Over the past four decades, research has successfully identified the meanings of many single calls across diverse taxa [6–18]. Building on this foundation, further research has shown that vocal sequences can also encode semantic content [19, 20], likely reflecting evolutionary pressures to convey ecologically and socially relevant information [21–24]. Studies showed that call combinations are commonly used across a range of vertebrate species to signal the presence of predators [25–30]. While much research has focused on alarm call combinations, sequences produced during other social contexts (e.g. greeting, travel coordination, food-related communication) remain less explored (but see recent work in chimpanzees *(Pan troglodytes)* [31–34], monkeys [35–37], meerkats *(Suricata suricatta)* [38] and elephants *(Loxodonta cyclotis)* [39]).

Animal vocal sequences can convey meaning through different mechanisms. A key distinction is whether a sequence is compositional or non-compositional [40]. In compositional sequences, the meaning of the sequence is derived from the meaning of at least one of the individual calls, such that the combination modifies or specifies the information conveyed compared to each call in isolation. In contrast, non-compositional sequences carry a meaning distinct from their composing units [33, 35, 41]. Furthermore, structural rules can influence meaning: the order in which calls are produced (e.g. AB vs. BA) and adjacent or non adjacent repetitions (i.e., iterations) of call types within sequences can shift meaning, as documented across species including birds, apes, monkeys and frogs [28, 29, 33, 42–49]. These rules are not mutually exclusive.

Identifying such mechanisms has traditionally relied on time-consuming playback experiments [19, 50], which are critical for assessing receiver comprehension but typically test one combination at a time. An approach better suited for assessing multiple vocal sequences uses observational data to analyse vocal sequences across an entire repertoire along with their contexts of production. To date, few wild species have been investigated this way, primarily apes [33, 42], though recent work has included meerkats and sperm whales *(Physeter macrocephalus)* [51, 52]. Such quantitative whole-repertoire analyses are crucial for understanding the evolutionary processes underlying expansive meaning generation, whereby comparative studies across species, including ape and non-ape species, can help assess whether flexible combinatorial capacities are evolutionarily ancient or represent more recent developments in the hominid lineage.

Sooty mangabeys *(Cercocebus atys)* provide an ideal comparative model for investigating combinatorial capacities in alarm and other social contexts in a non-ape species. Like chimpanzees and bonobos *(Pan paniscus)*, they inhabit dense African rainforests, form large multi-male, multi-female groups of up to 100 individuals and are highly terrestrial [53–56]. This convergent ecology presents overlapping communicative challenges: maintaining cohesion and navigating social relationships within large groups in visually obstructed habitats and potentially responding to multiple predator types [57–63]. Testing whether sooty mangabeys exhibit combinatorial capacities within social contexts can help determine whether such abilities are evolutionarily ancient or represent more recent hominid adaptations.

In our study, we investigated vocal sequence production across the full vocal repertoire of sooty mangabeys, a forest-dwelling monkey species known to produce call sequences [64, 65], acknowledging that constraints in behavioural data collection may prevent the recording of all possible utterances. To do so, we used the context of production as a proxy for potential meaning [13, 57, 66–69]. Context referred to the set of features characterising the circumstances surrounding the production of each utterance [19], also called ‘events’ in other studies [33]. In our study and following the framework proposed by Berthet et al. 2023, contextual features include not only what is commonly referred to as context such as the type of activity (e.g. feeding) or the social interaction (e.g. affiliation) but also the attributes of the interaction partner (i.e. age class and/or sex). This was important as sooty mangabeys often vocalise during social interactions, and the usage of specific call types appears to vary depending on partner characteristics [58, 70].

Adult sooty mangabeys can combine most of their call types in at least one combination (eight out of ten call types, with only ‘vibratos’ and ‘whoop-gobbles’ never recorded as part of a sequence), but they frequently rely on a limited subset of combinations, typically sequences composed of ‘grunt’ and ‘twitter’ calls or ‘shrill’ and ‘hoo’ calls [64]. In particular, sequences composed of ‘grunt’ and ‘twitter’ calls are only produced by females either as simple bigrams (e.g. ‘grunt_twitter’, ‘twitter_grunt’) or as longer sequences with iterations (e.g. ‘grunt_twitter_grunt’, ‘twitter_grunt_twitter_grunt’), which raises the question of whether both order and/or iteration may influence meaning [64]. See the glossary for our definitions of call, bigram, sequence and iteration.

We therefore aimed to determine whether sooty mangabey call sequences are compositional, that is, whether the meaning can be derived from the calls produced individually, but also whether ordering and iterations are associated with the potential meaning of the sequence. To investigate this, we focused on:

(1) Bigrams (i.e. sequences composed of two distinct call types) following an analytical approach recently applied to a full repertoire analysis of chimpanzee vocal sequences [33]. This approach allowed us to describe to what extent bigrams exhibited compositional or non-compositional structures, and whether the associated contextual features composing the context of production were associated with the order of calls.

(2) Two-call-type sequences with iterations, to examine whether they differed in potential meaning from their component calls and bigrams, and whether the order of calls within these iterative sequences also influenced potential meaning (e.g. whether A_B_A differs from A, B, A_B, and/or B_A_B).

A French translation of the abstract is provided in Additional file 1.

## Glossary

Following previous work on sooty mangabey sequences [64, 65], we used the following definitions:

**Table:** 

Term	Definition
Vocal element	Continuous sound with a distinct beginning and end.
Call type	One or more vocal elements of the same type produced within an interval of less than two seconds. A call type can occur singly or as part of a sequence. For example, multiple ‘grunt elements’ produced in quick succession (e.g. grunt-grunt-grunt) constitute one instance of a 'grunt' call type. These criteria limit over-representation of calls in the dataset [64, 65]
Sequence	Two or more different call types produced with intervals of less than one second between them (e.g. A_B, A_B_A). A sequence always contains at least two distinct call types. These criteria limit over-estimation of sequences in the dataset [64, 65]
Bigram	A specific type of sequence composed of exactly two different call types (e.g. A_B). A bigram can occur as a complete sequence by itself or as part of a longer sequence (e.g. the A_B portion within A_B_A)
Iteration	The non-adjacent reoccurrence of a call type within a sequence, interspersed with other call types (e.g. in A_B_A, call type A iterates). Sequences with iteration contain at least three calls
Vocal utterance	The broadest term, encompassing either a single call type produced in isolation or a sequence of different call types

## Results

Our dataset comprised *N* = 2023 vocal utterances, including *N* = 1751 for which we could identify the set of contextual features associated with their production.

### Females—utterance types recorded

We recorded 1501 utterances produced by females where we could document a clear context of production, including 1245 single calls, 144 bigrams, and 112 sequences longer than two calls (range, 3–38 calls; sequences comprising more than five calls were rare, occurring fewer than ten times). The most frequent utterance type was ‘grunt’ (55.9%), followed by ‘twitter’ (7.5%), ‘vibrato’ (6.7%), ‘grunt_twitter’ (4.8%), ‘grunt_twitterX’ (4.1%), ‘growl’ (3.8%), ‘scream’ (2.7%), ‘twitter_grunt’ (2.7%) and ‘twitter_gruntX’ (1.9%). All other utterance types occurred fewer than nine times (see Additional file 2: Tables S1-S2 for full details on sample sizes).

Overall, the main female sequences were composed exclusively of ‘grunt’ and ‘twitter’ calls (*N* = 215 out of 257 sequences; 84%). These included the bigrams ‘grunt_twitter’ (*N* = 73, 26 individuals) and ‘twitter_grunt’ (*N* = 43, 17 individuals) or iterative forms—‘grunt_twitterX’ (*N* = 65, 18 individuals) and ‘twitter_gruntX’ (*N* = 30, 11 individuals) (see Additional file 2: Tables S1-S2 for complete breakdowns).

### Females—contextual usage of single calls

**Fig. 1:**
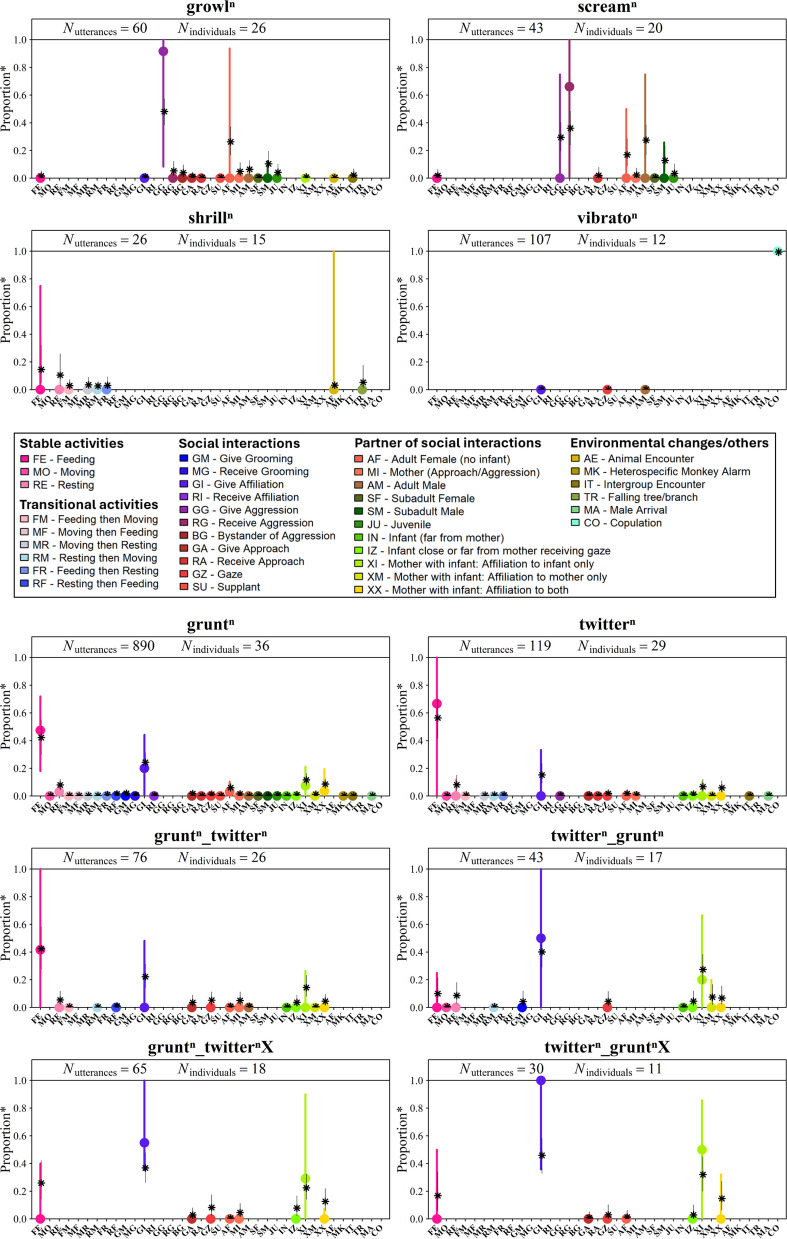
Distribution of contextual features of the most frequently recorded female vocal utterances. Coloured dots show the observed median individual proportion of occurrence of each utterance in each contextual feature (50% of individuals ≥ *P*). Vertical coloured lines indicate the 25th percentile (75% of individuals ≥ *P*) and 75th percentile (25% of individuals ≥ *P*). Black stars and thin black lines show the posterior median and 89% credible interval estimated by our statistical model. Superscript *n* indicates that each call in the vocal utterances may include any number of vocal element repetitions (from zero upwards). Note: “Proportion*” refers to values calculated per contextual feature and may sum to more than one across all features because some utterances were assigned to multiple contextual features. For example, when a single utterance involved a social partner, it was counted for both the type of social interaction and recipient attribute (e.g. *give affiliation* + *adult female*). Each individual proportion remains ≤ 1, but the total across contextual features may exceed one as utterances can contribute to multiple feature types

The observed distribution of vocal utterances across contexts is shown in Fig. 1. Female ‘growls’ were predominantly observed when giving aggression (GG) to other adult females (AF), ‘screams’ most frequently when receiving aggression (RG), ‘shrills’ during feeding (FE) and when encountering animals other than monkeys (AE) and ‘vibrato’ calls almost exclusively during copulation (CO).

Female ‘grunts’ were produced across a variety of contextual features but occurred most frequently during feeding (FE) and when giving affiliations (GI) that could be directed at infants only (XI) or both the mother-infant pair (XX), although in low proportions (< 10%; Fig. 1 and Table 1). These same contextual features also accounted for most occurrences of single ‘twitters’ (a call type not produced by adult males), with the addition of resting (RE) (Fig. 1). Euclidean distances confirmed that ‘grunt’ and ‘twitter’ were contextually more similar to each other than to any other single call type (Additional file 3: Figure S1).

**Table 1:** Most frequent contextual features in which ‘grunt’ and ‘twitter’ calls were produced both singly and in sequences. Values in bold highlight the type of activity and social interaction (either ‘feeding’ or ‘give affiliation’) in which the highest number of utterances was recorded. When ‘give affiliation’ is the most frequent, bold values indicate the most frequent type of recipient of affiliation (‘to infant only’ = affiliative behaviours directed at infant carried by or close to their mother, ‘to infant + mother pair’ = affiliative behaviours directed at both an infant and their mother). Numbers on top within each cell represent the observed median individual proportion of each utterance type in a given contextual feature, with the 25% and 75% quartiles in brackets. Values in italics below indicate the median proportion from the model’s posterior distribution, with the 89% credible interval in brackets

Utterance type	Feeding	Give affiliation	To infant only	To infant + mother pair
grunt	**0.47 [0.18–0.72]** ***0.42 (0.30–0.55)***	0.20 [0.0–0.44] *0.24 (0.18–0.31)*	0.7 [0.0–0.21] *0.12 (0.8–0.16)*	0.4 [0.0–0.20] *0.8 (0.5–0.13)*
twitter	**0.67 [0.0–1]** ***0.56 (0.42–0.70)***	0.0 [0.0–0.33] *0.15 (0.9–0.23)*	0.0 [0.0–0.10] *0.7 (0.3–0.12)*	0.0 [0.0–0.0] *0.6 (0.3–0.11)*
grunt_twitter	**0.42 [0.0–1]** ***0.42 (0.28–0.58)***	0.0 [0.0–0.48] *0.22 (0.14–0.31)*	0.0 [0.0–0.26] *0.14 (0.8–0.23)*	0.0 [0.0–0.0] *0.5 (0.2–0.10)*
twitter_grunt	0.0 [0.0–0.25] *0.10 (0.4–0.22)*	**0.50 [0–1]** ***0.40 (0.29–0.52)***	**0.20 [0.0–0.67]** ***0.27 (0.17–0.39)***	0.0 [0.0–0.20] *0.7 (0.2–0.16)*
grunt_twitterX	0.0 [0.0–0.40] *0.26 (0.14–0.42)*	**0.55 [0.35–1]** ***0.37 (0.26–0.48)***	**0.29 [0.0–0.90]** ***0.22 (0.14–0.33)***	0.0 [0.0–0.8] *0.13 (0.6–0.22)*
twitter_gruntX	0.0 [0.0–0.50] *0.17 (0.60–0.34)*	**1 [0.36–1]** ***0.46 (0.33–0.58)***	**0.50 [0.0–0.96]** ***0.32 (0.20–0.45)***	0.0 [0.0–0.32] *0.15 (0.6–0.27)*

### Contextual shifts in female ‘grunt’ and ‘twitter’ sequences

Given sufficient sample sizes for quantitative analysis, we focused on sequences composed of ‘grunt’ and ‘twitter’ calls to examine whether call order and iteration were associated with contextual shifts in usage.

### Ordering effect in bigrams

**Fig. 2:**
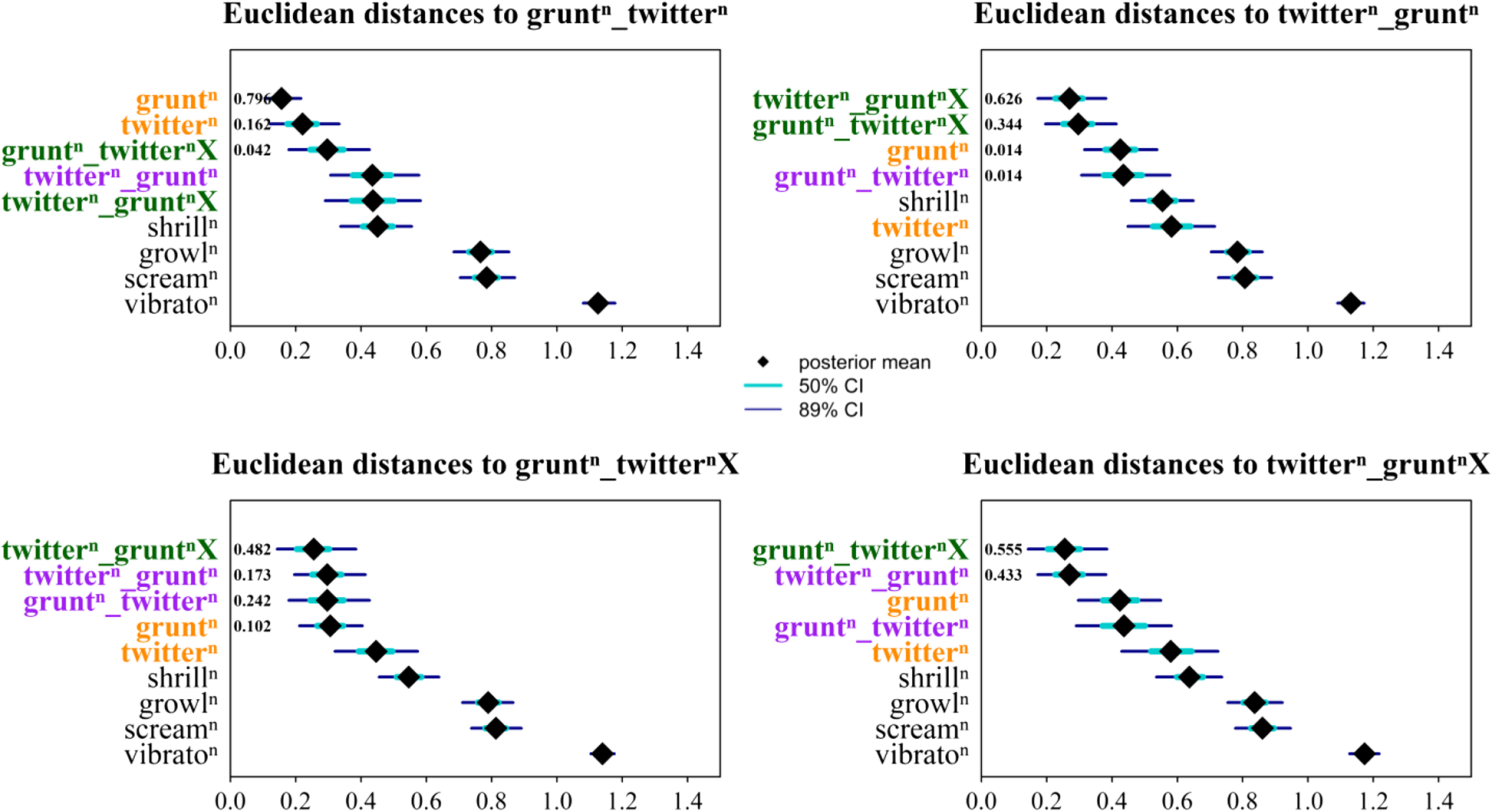
Euclidean distances measuring contextual similarity between sequences and other common vocal utterances. For each sequence, the mean distance and the 50% and 89% credible intervals relative to each comparison utterance are shown. Component single calls are displayed in orange, component bigrams in purple and iterative sequences in green. *Y*-axis values indicate the proportion of posterior samples in which the corresponding utterance was the closest match to the focal sequence (p.support); only values greater than 0.01 are displayed. Superscript *n i*ndicates that each call in the vocal utterances can have any number of vocal element repetitions (from zero upwards)

**Fig. 3:**
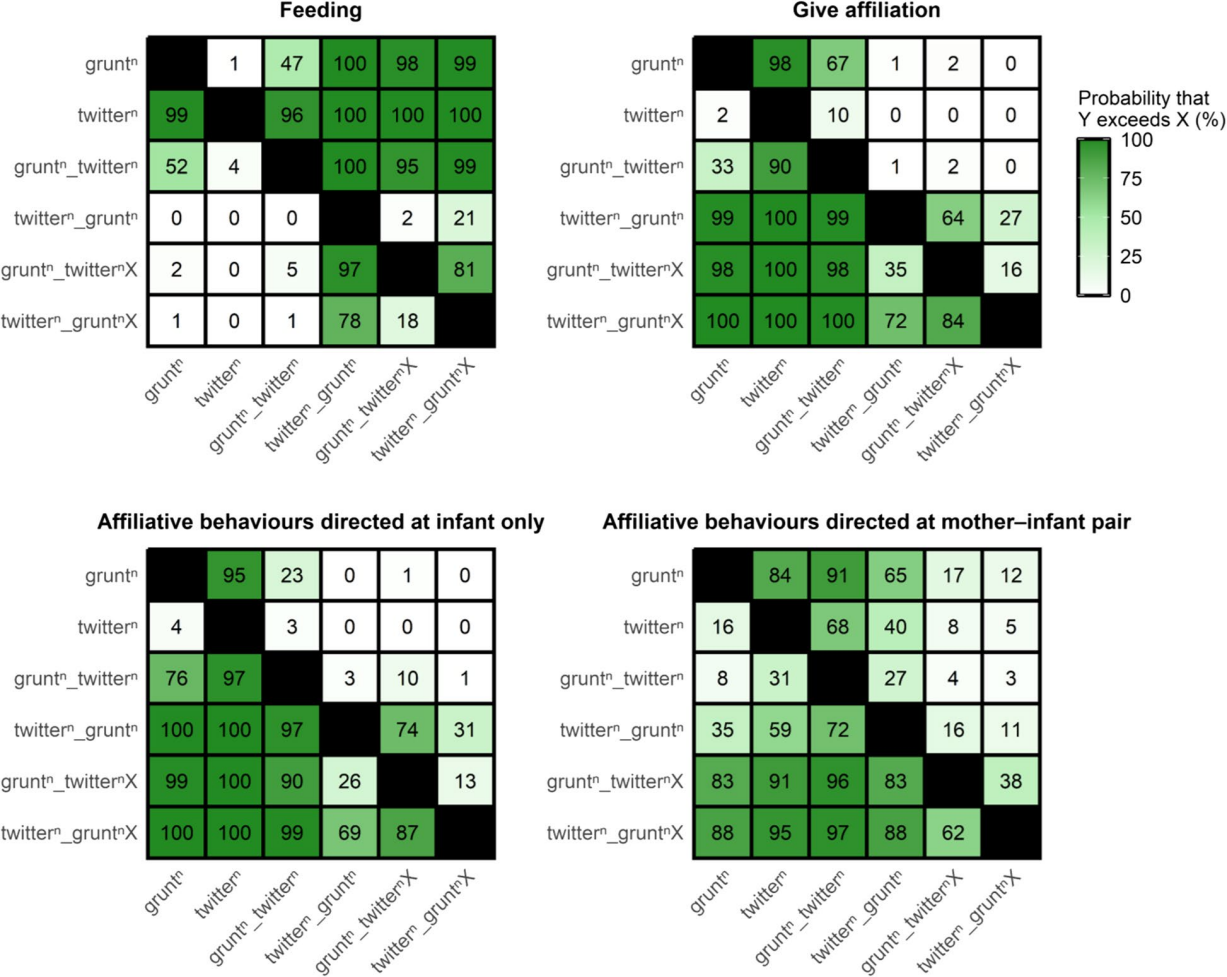
Pairwise comparisons of the posterior distributions of the proportion of utterance occurrences produced in each contextual feature. Each cell shows the percentage of posterior samples in which the utterance on the *Y*-axis was estimated to occur more frequently in the specified contextual feature (see matrix titles) than the utterance on the X-axis. Forest green shades indicate the strength of difference, with darker green representing higher percentages. Numbers inside cells are the rounded percentage values. Superscript *n* indicates that each call in the vocal utterances can have any number of vocal element repetitions (from zero upwards)

Among bigrams, ‘grunt_twitter’ followed a similar distribution to its component calls, being used primarily during feeding (FE) and less so when giving affiliation (GI) (Fig. 1 and Table 1). This was also supported by Euclidean distances indicating that the bigram ‘grunt_twitter’ was close in terms of context distribution to its component calls (Fig. 2 and Table 2). In contrast, ‘twitter_grunt’ was clearly more frequent when giving affiliation (GI), especially when directed towards infants (XI and XX), and appeared less often uttered while feeding (FE) (Fig. 1 and Table 1). Here, Euclidean distances showed less similarity to the contextual distribution of the component calls (Fig. 2 and Table 2). This was further supported by the pairwise comparisons of the posterior distributions of the proportions of vocal utterances during feeding, affiliative, and infant-directed contexts between vocal sequences and their constituent calls. We found 100% posterior support for the proportion of feeding to be higher when uttering single ‘twitters’ and single ‘grunts’ than when uttering the bigram ‘twitter_grunt’ (i.e. very strong evidence that the sequence is used less during feeding than its component calls). Likewise, we found 99–100% posterior support for ‘twitter_grunt’ to be more often uttered during affiliation and directed at infants than single ‘grunts’ and single ‘twitters’ (i.e. very strong evidence that the sequence is used more during affiliation involving infants than its component calls) (Fig. 3).

**Table 2:** Euclidean distances between each target sequence and closest utterances, based on differences in contextual feature distribution. The dominant contextual feature (i.e. most frequent contextual feature of production) is indicated for both target sequences and closest utterances. p.support indicates the proportion of posterior samples in which a given utterance was identified as the closest to the target sequence in terms of Euclidean distance. Only utterances with a p.support value greater than 0.01 are shown. Distances were calculated using all main utterance types (‘grunt’, ‘grunt_twitter’, ‘grunt_twitterX’, ‘twitter’, ‘twitter_grunt’, ‘twitter_gruntX’, ‘shrill’, ‘growl’, ‘scream’, ‘vibrato’)

Target sequence	Target sequence dominant contextual feature	Closest utterances	Distance	p.support	Closest utterances dominant contextual feature
grunt_twitter	Feeding	grunt	0.16	0.80	Feeding
		twitter	0.22	0.16	Feeding
		grunt_twitterX	0.30	0.04	Affiliation with infant
twitter_grunt	Affiliation with infant	twitter_gruntX	0.27	0.63	Affiliation with infant
		grunt_twitterX	0.30	0.34	Affiliation with infant
grunt_twitterX	Affiliation with infant	twitter_gruntX	0.26	0.48	Affiliation with infant
		twitter_grunt	0.30	0.17	Affiliation with infant
		grunt_twitter	0.30	0.24	Feeding
		grunt	0.31	0.10	Feeding
twitter_gruntX	Affiliation with infant	grunt_twitterX	0.26	0.56	Affiliation with infant
		twitter_grunt	0.27	0.43	Affiliation with infant

### Comparing iterative vocal sequences to bigrams

Iterative sequences (‘grunt_twitterX’ and ‘twitter_gruntX’), regardless of whether they began with ‘grunt’ or ‘twitter’, showed greater contextual similarity to ‘twitter_grunt’ bigrams than to ‘grunt_twitter’ bigrams. Specifically, these sequences occurred more frequently during affiliative interactions (GI) with infants (XI and XX) than during feeding (FE) (Fig. 1 and Table 1). Euclidean distance analyses showed that ‘twitter_gruntX’ was produced in a very similar context to both ‘twitter_grunt’ and ‘grunt_twitterX’ yet remained more distant from its component calls (i.e. more dissimilar in terms of contextual usage). In contrast, ‘grunt_twitterX’ displayed a more intermediate pattern: it was contextually close not only to ‘twitter_gruntX’, but also to ‘twitter_grunt’, ‘grunt_twitter’, and ‘grunt’ (Fig. 2 and Table 2). Pairwise comparisons of the posterior distribution of the proportion of utterance types by contexts (Fig. 3) revealed:

- 97% and 81% posterior support for ‘grunt_twitterX’ to be more likely to occur during feeding than ‘twitter_grunt’ and ‘twitter_gruntX’, respectively.
- ‘grunt_twitterX’ was less likely to occur when giving affiliations directed at infants than ‘twitter_grunt’ and ‘twitter_gruntX’, but these differences were supported by less than 85% of the pairwise posterior distribution comparison (Fig. 3).

Thus, ‘grunt_twitterX’ iterative sequences were more similar to ‘twitter_grunt’ bigrams and ‘twitter_gruntX’, but were generally preferred during feeding and less so during infant-directed affiliations by comparison.

### Recipient of affiliation

We could record with certainty the recipient for all vocal utterances used during affiliative interactions. Those directed only towards infants were clearly dominated by ‘twitter_grunt’ and ‘twitter_gruntX’ sequences (Figs. 1 and 3). In contrast, when affiliation was directed at both the infant and the mother, the distribution was more balanced. We found between 83 and 100% posterior support for iterative sequences to be uttered more often in these types of affiliation than bigrams or single calls (i.e. moderate to strong evidence that iterative sequences are preferentially used in affiliative contexts compared to bigrams). Notably, single ‘grunts’ were also more likely than the ‘twitter_grunt’ bigram, but with weak posterior support (65%) for this difference (Figs. 1 and 3). Generally, while single ‘grunts’ were produced towards a broader range of recipients, including adult females without infants (*N* = 12 ‘grunts’ from 10 individuals), they were still more frequently directed at females with infants, similar to single ‘twitters’ and their combinations (Fig. 1 and Additional file 2: Table S3 for details).

### Males—utterance types recorded

In the *N* = 249 male vocal utterances with identified contexts of production, we recorded *N* = 213 single calls, *N* = 21 bigrams and *N* = 15 sequences longer than two calls (ranging from three to six calls). Among males, the predominant single calls with a clear production context were ‘grunts’, accounting for 83.6% of single calls, followed by ‘growls’ at 16%. The most frequently observed bigram was ‘shrill_hoo’ (*N* = 19), produced by three individuals. All other single calls and sequences (bigrams or longer) occurred five times or fewer (see Additional file 2: Table S4 for complete sample sizes).

### Males—contextual usage of single calls and bigram

**Fig. 4:**
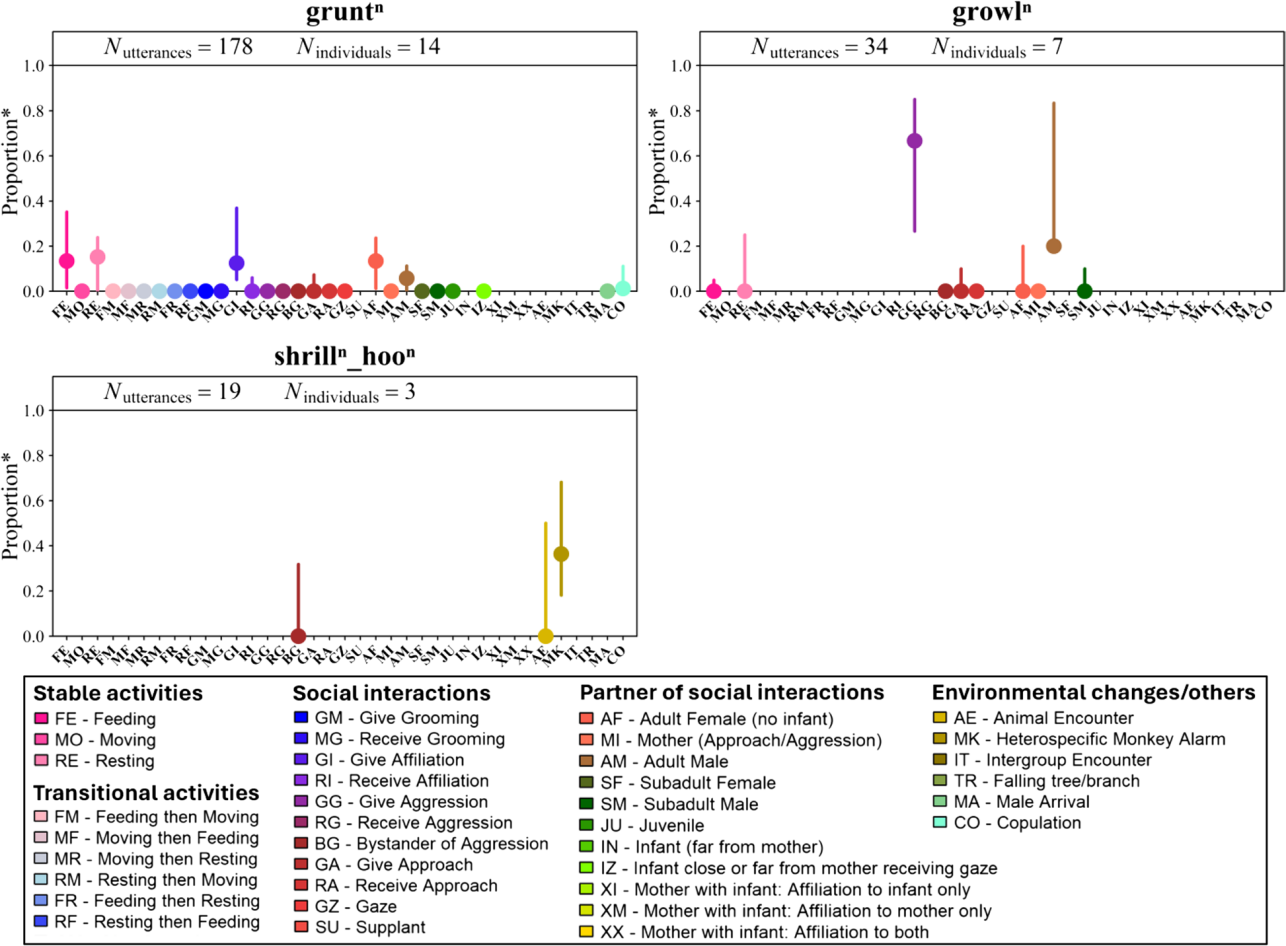
Distribution of contextual features of the most frequently recorded male vocal utterances. Coloured dots show the observed median individual proportion of occurrence of each utterance in each contextual feature (50% of individuals ≥ *P*). Vertical coloured lines indicate the 25th percentile (75% of individuals ≥ *P*) and 75th percentile (25% of individuals ≥ *P*). Superscript *n* indicates that each call in the vocal utterances may include any number of vocal element repetitions (from zero upwards). Note: “Proportion*” refers to values calculated per contextual feature and may sum to more than one across all features because some utterances were assigned to multiple contextual features. For example, when a single utterance involved a social partner, it was counted for both the type of social interaction and recipient attribute (e.g. *give affiliation* + *adult female*). Each individual proportion remains ≤ 1, but the total across contextual features may exceed one as utterances can contribute to multiple feature types

Like females, male ‘grunts’ occurred across a wide range of contextual features, with higher proportions recorded during feeding (FE), resting (RE) and affiliative interactions (GI), particularly those directed at adult females (AF) and other adult males (AM) (Fig. 4). Note that males do not produce ‘twitters’. Male ‘growls’ were mostly produced during aggressive interactions (GG), especially towards other adult males (AM). ‘Shrill_hoo’ bigrams were more frequently given in response to monkey alarm calls (MK), and occasionally during encounters with non-monkey animals (AE) or as a bystander to aggression (BG) (Fig. 4).

## Discussion

We used a whole-repertoire approach to assess vocal combinatorial usage across the vocal repertoire and daily life of sooty mangabeys. Vocal sequences were predominantly emitted by females combining two call types, ‘grunts’ and ‘twitters’. These sequences were emitted in sufficient sample sizes for quantitative analysis of meaning shifts. Single ‘grunts’ and ‘twitters’ occurred primarily during feeding and, to a lesser extent, affiliative contexts involving infants. When combined into sequences, the bigram ‘grunt_twitter’ remained primarily associated with feeding, similar to its component calls. However, call order produced a dramatic usage shift for the bigram ‘twitter_grunt’, which was predominantly used during infant-directed affiliation. Iterative sequences (‘twitter_gruntX’ and ‘grunt_twitterX’) showed similar contextual patterns to ‘twitter_grunt’, with minor variations depending on which call initiated the sequence. In adult males, which do not emit ‘twitters’, limited vocal sequence emissions precluded comparable analyses, though we descriptively report a male proclivity to emit the ‘shrill_hoo’ vocal sequence during alarm contexts.

Before examining vocal sequence usage, we confirmed that the contextual use of single calls across both sexes aligns well with descriptions from the previously established vocal repertoire, which focused solely on single calls (Range & Fischer, 2004). Below, we discuss the patterns and potential shifts in meaning observed in the vocal sequences.

### Females—contextual usage of vocal sequences

Vocal sequences produced by females predominantly included just two call types, used in various configurations: ‘grunts’ and ‘twitters’. When emitted singly, these calls overlapped contextually, primarily during feeding and affiliation involving infants, with feeding as the dominant context (Fig. 1 and Table 1). These calls were combined into bigrams, in either order, and into iterative sequences, which allowed us to address our main research question: whether combining calls into sequences produces meaning shifts through ordering and iteration.

### Ordering effect in bigrams

Our results show that combining two potentially meaning-bearing calls can lead to context-specific shifts in usage, depending on production order. Single ‘grunts’, ‘twitters’, and ‘grunt_twitter’ bigrams were mainly associated with feeding, and to a lesser extent, affiliative interactions involving infants (Fig. 1 and Table 1). In contrast, the ‘twitter_grunt’ bigram showed a pronounced contextual shift, as it was almost exclusively used in affiliative contexts with infants (Fig. 1 and Table 1). This order-dependent contrast suggests that while the individual calls have overlapping meanings, combining them in specific orders can clarify or narrow meaning.

Because both bigrams and single calls occurred during similar contexts but with different probabilities, we can rule out idiomaticity, i.e. sequence meaning unrelated to component calls [41, 71]. Instead, our findings suggest order-sensitive compositionality with clarification: the sequence meaning derives from the meaning of its components, but this depends on which call initiates the sequence [33]. Ordering effects have been described in chestnut-crowned babbler *(Pomatostomus ruficeps)* and olive colobus monkey *(Procolobus verus)* sequences, though in those cases, the calls are not produced in isolation, so their meanings cannot be traced to individual components, which limits evidence of compositionality [29, 72]. In Campbell’s monkeys *(Cercopithecus campbelli)*, sequences like ‘boom_krak’ are biased toward the meaning of the first call (‘boom’, a non-urgent threat), aligning with the “urgency principle” where critical information is produced first [73, 74]. In this sense, ‘twitter_grunt’ appears more comparable to order-sensitive bigrams in chimpanzees, which are produced in feeding and social interaction contexts [33], or to the ‘alert–recruitment’ sequences of Japanese tits *(Parus minor)*, to which receivers respond only when presented in that specific order and not the reverse [47, 75].

Compositionality involving non-alarm calls is rarely reported outside of great apes [33, 42], but see recent work in elephants [39]. More commonly, bigrams in socio-positive contexts feature affix-like elements added to stand-alone calls (e.g. Diana monkeys *(Cercopithecus diana)* [36], Campbell’s monkeys [37], mongooses *(Mungos mungo)* [38]), typically conveying signaller identity rather than refining context-specific meaning. Given that ‘twitter_grunt’ bigrams overwhelmingly target infants, identity-related information may be encoded in the calls so that infants learn group member calls. However, if identity marking were the sole function, we would not expect ‘twitter_grunt’ to shift so dramatically toward infant-directed affiliation while its component calls and the reverse bigram ‘grunt_twitter’ remain primarily associated with feeding contexts. This shift in contextual distribution indicates that the sequence meaning is derived from its components in an order-sensitive manner, possibly encoding information about the social interaction rather than marking caller identity through affixation. Alternatively, it may signal benign intent as bigram emission is often associated with infant affiliation. In baboons, approaches to mothers with infants which are accompanied by ‘grunts’ compared to without ‘grunts’ more likely result in infant handling and have been interpreted as a signal of benign intent in an otherwise potentially threatening situation, as adults can harm infants [76]. Overall, our findings suggest a case of compositionality in a socially benign context, specifically during affiliation involving infants.

### Comparing iterative vocal sequences to bigrams

Iterative sequences showed contextual patterns largely similar to ‘twitter_grunt’ bigrams, addressing our second research question about whether iteration modulates meaning. ‘Twitter_gruntX’ was predominantly used in infant-directed affiliative contexts, suggesting iteration extends ‘twitter_grunt’ without fundamentally shifting meaning (Figs. 1 and 3, Tables 1 and 2). ‘Grunt_twitterX’ showed an intermediate pattern, occurring in both feeding and affiliative contexts (Figs. 1 and 3), suggesting that starting with ‘grunt’ broadens contextual usage compared to starting with ‘twitter’.

Such patterns raise the possibility that the number of occurrences of a specific call type within a sequence could modulate meaning. Indeed, in several species, including bonobos [44, 77], chickadees *(Poecile carolinensis)* [28], great tits *(Parus major)* [46], putty-nosed monkeys *(Cercopithecus nictitans)* [35], titi monkeys *(Callicebus nigrifrons)* [25] and frogs [43], the number of repetitions (whether adjacent or not) of specific call types or vocal elements within sequences has been linked to semantic content. However, these studies typically report the overall number of occurrences without specifying whether these were consecutive repetitions of adjacent vocal elements composing a call (as defined in our study) or iterations of call types interspersed with other call types within a sequence (e.g. A_B_A). Nevertheless, they highlight that patterns of call reoccurrence within sequences likely contribute to meaning.

Interestingly, ‘grunt_twitterX’ sequences appear more likely to end with ‘grunt’ calls [64]. One potential hypothesis is that these sequences are composed of one initial ‘grunt’ followed by one or more ‘twitter_grunt’ bigrams. Under this hypothesis, this may indicate that the caller embeds an infant-directed or infant-related message (‘twitter_grunt’) within a broader feeding context, clarified by the terminal ‘grunt’. In contrast, ‘twitter_gruntX’ does not appear to show a consistent terminal pattern [64], possibly because starting with ‘twitter’ already signals infant-directed affiliation. However, the idea that bigrams are embedded into iterative sequences remains speculative and warrants further investigation.

### Recipient of affiliation

Given the variation in utterance usage within affiliative contexts, we explored whether the recipient influenced this variation. Female single ‘grunts’ in affiliation mainly were directed toward infants near or carried by their mothers, and less frequently towards other individuals. Conversely, male ‘grunts’ tended to target adult females without infants or other adult males (Fig. 4). This likely reflects sex differences in social roles, with females being particularly interested in affiliative interactions involving infants [78]. This pattern was more apparent in females when calls were combined into ‘twitter_grunt’, ‘twitter_gruntX’ and ‘grunt_twitterX’ sequences. Notably, when affiliative behaviours were directed solely at infants, females mainly used ‘twitter_grunt’ and ‘twitter_gruntX’. When directed at both mothers and infants, iterative sequences were more common, suggesting the recipient influences call combination use.

In many primates, calls before or during infant interactions signal benign intent and may facilitate access to infants by reducing maternal aggression [79–81]. For instance, in chacma *(Papio ursinus)* and olive *(Papio anubis)* baboons, vocal production during infant handling is influenced by kinship and dominance between handler and mother [76, 82], and Guinea baboons *(Papio papio)* produce more ‘grunts’ when social bonds with mothers are weak [83]. In sooty mangabeys, females handle infants gently, vary in interest based on infant age and availability, and show weaker kin biases than other primates [78]. Grooming is often used to gain access, especially by lower-ranking females [78]. Importantly, calls may also be directed at infants [84]. In rhesus macaques *(Macaca mulatta)*, handlers use two distinct call types during infant handling: ‘girneys’, which may attract infant attention specifically, and ‘grunts’, which are produced in many contexts and may simply signal harmless intent [85]. Thus, sooty mangabey females may use single calls, bigrams or iterative sequences, depending on factors like interest in the infant, caller–mother rank difference, kinship and whether the utterance is directed at the infant, the mother or both. These findings suggest that utterances in infant-directed affiliations may encode specific information about social negotiation or infant-directed communication [84]. Further research is needed to clarify these patterns.

### Males– contextual usage of vocal sequences

Adult males regularly produced one vocal sequence in our dataset, the ‘shrill_hoo’ bigram, although its components were not observed in isolation. This pattern is consistent with previous findings reporting lower sequence diversity in males compared to females [65]. While ‘hoo’ calls are rarely produced alone, ‘shrill’ calls occur independently in females. The absence of isolated ‘shrill’ calls in males prevented us from comparing bigrams to components. Instead, we focused on the contextual features of ‘shrill_hoo’, which was mostly produced during animal encounters and in response to heterospecific alarm calls (Fig. 4), consistent with its role in signalling threats [60, 70]. It also occurred, though less frequently, when callers witnessed conspecific aggression, suggesting a broader communicative function. This raises the possibility of acoustic variants by context, as in chacma baboons [86], but confirming this would require further research.

### Whole repertoire insights

Contexts of sooty mangabey vocal production, such as affiliation, aggression and alarm, overlap with those in many other animals [87]. Call combinations in sooty mangabeys likely function to expand messaging precision, particularly in socially benign contexts. Vocal sequences also occurred in alarm contexts and during aggression, although the sample size during aggression was too small to assess where sequence structure shifted meaning compared with the component calls. Overall, our findings fit with a major hypothesis that social complexity drives the evolution of complex communication [21–23, 88, 89]. Our findings fit with recent studies demonstrating that vocal sequence emissions are promoted by two contexts which create particular communication challenges in complex societies: social uncertainty (here, surrounding aggression) or coordination challenges (here, coordinating affiliation with others’ infants) [58, 59]. Grampp et al. demonstrated that within and between species (chimpanzees and sooty mangabeys), signal sequences are more likely to occur in socially uncertain or in challenging coordination contexts. Outside of primates, the few studies demonstrating compositional-like rule-based vocal sequences are also emitted during contexts that require coordination, specifically alarm + recruitment situations (e.g. Japanese tits [75]). We add to this, demonstrating that compositional-like structural rules in sooty mangabeys may be promoted by coordination challenges.

Whilst we clearly demonstrate use of call combinations outside of alarm contexts, the capacity to combine single calls into context-dependent bigrams appears less versatile in sooty mangabeys compared to chimpanzees [33] and bonobos [42]. Still, whether this reflects a hominid-specific ability to flexibly use compositional-like structures across daily life situations remains unclear, as comparable whole-repertoire analyses are still rare for other monkeys and non-primate species. Broader comparative work across taxa will be crucial to determine the extent to which such combinatorial capacities are shared or represent lineage-specific capacities.

### Limitations

Despite careful observation, certain vocal utterances were under-represented in the dataset due to data collection limitations. This was particularly the case during fast-paced interactions, such as aggressive encounters within or between groups, where multiple individuals often vocalised simultaneously, or during alert events when it was not possible to identify the cause of the alert. Other contexts where vocalisations may have been produced, such as in tall trees or at night, when the caller could not be seen, could also not be reliably recorded. In addition, our categorisation of calls into broad types may have overlooked finer acoustic variation within each category. For example, ‘twitters’ may vary acoustically depending on the context of production [70]. If such variation carries context-specific meaning, it could influence the interpretation of utterances, an aspect not addressed in our analyses. We also did not examine the potential roles of adjacent repetitions of vocal elements within call types or the number of call iterations in sequences composed of ‘grunt’ and ‘twitter’, both of which potentially contribute to utterance meaning. Finally, playback experiments are necessary to determine the function of these call combinations, and the meaning conveyed to receivers and third parties.

## Conclusions

Our study demonstrates that sooty mangabeys combine singly-used, potentially meaning-bearing calls into sequences. Focusing on vocal sequences combining ‘grunts’ and ‘twitters’ calls (as sooty mangabeys emit these sufficiently frequently to allow us to use quantitative analysis), we found that contextual usage (i.e. feeding vs. infant-directed affiliation) influenced call combinations used, call order within sequences, and call iteration. Specifically, while single calls largely aligned with previously described contextual associations, bigrams, especially ‘twitter_grunt’, showed a contextual shift in usage, suggesting a form of compositionality shaped by ordering. Iterative sequences further indicate that meaning may be preserved or broadened depending on which call initiates the sequence, with some patterns hinting at the possibility that embedding of bigrams into longer iterated sequences may impact messaging. These findings contribute to the growing body of evidence that nonhuman animals can utilise rule-based vocal combinations to refine their communication during social interactions and expand their messaging beyond what is possible with single calls alone. The contexts of usage of vocal sequences across the repertoire fit with a key hypothesis that social complexity drives complex communication. Specifically, rule-based vocal sequence usage was evident in a delicate social situation requiring coordination to facilitate adult females approaching mothers to affiliate with their infants. Future research should also investigate how recipient attributes, such as caller–receiver kinship and social rank, influence call combination usage, and importantly, whether these vocal sequences convey different meanings to receivers.

Crucially, we propose that compositionality in socio-positive contexts might not be restricted to apes but may also be present in monkeys, suggesting that this shared capacity is evolutionarily ancient. However, while sooty mangabeys might exhibit this context-sensitive combinatorial capacity, they do not demonstrate the broad and flexible use of compositional-like structures across daily life seen in *Pan* species. Whether such extensive combinatorial capacities represent a hominid-specific adaptation remains an open question, largely because comparable whole-repertoire analyses are scarce, not only in monkeys but also more generally across non-primate taxa. Thus, more systematic and comparative research across diverse animal taxa is essential to clarify the evolutionary distribution of these communicative abilities and to understand where similar or divergent capacities might lie.

## Methods

### Data collection

For this study, ALF, NB and TSA collected vocal and behavioural data on two groups of sooty mangabeys: the Taï Monkey Project (TMP) group (May to July 2022) and the Taï Chimpanzee Project (TCP) group (February to October 2023) in the Taï National Park, Ivory Coast (McGraw et al., 2007; Wittig, 2018). Individuals were fully habituated to human presence, allowing close range observation and audio recording (1–10 m). All three observers completed training in individual identification of sooty mangabeys prior to data collection. We employed half-day continuous focal animal sampling [90] on adult individuals, conducting observations from dawn to midday and from midday to dusk. During these sessions, we recorded focal individual vocalisations, as well as calls from known adult individuals within visual range ad libitum, provided we could clearly determine caller identities. Based on prior familiarity with the vocal repertoire and training with experienced field assistants, we were able to make initial in-field identifications of most call types. To facilitate later identification and coding of focal individual calls in the recordings, we provided spoken notes to verbally indicate the presumed call types. Formal inter-observer reliability tests were conducted subsequently to confirm consistent classification (see section “audio recording coding” below). When other individuals produced calls, we verbally indicated them as well to avoid mistakenly coding non-focal calls later. Recordings were made using directional microphones (Sennheiser models K6, MK 316, MKH 416 P48, and MKH 416 T-F) and Marantz Solid State recorders (PMD661 and PMD661 MKII), digitised at a 44.1 kHz sampling rate and 16-bit depth in.wav format.

For each recorded vocalisation, we noted the features of the context of production (i.e. the contextual features, defined as the set of features surrounding the vocal production) using a predefined ethogram (see Supplementary Table S1), based on previously published work on sooty mangabey behaviours and contexts eliciting vocalisations [58, 60, 62, 70, 91–95], and recorded using Cybertracker on a smartphone.

### Contextual features

We assessed 37 contextual features associated with vocal production, as detailed in Additional file 4: Table S5. These included social interactions, defined as beginning when one individual initiated gaze or approached another, and ending when they moved apart or ceased social behaviours and gazing. When a vocal utterance occurred during such an interaction, we assigned two contextual features: [1] the type and direction of the social interaction involving the caller (e.g. give approach, give affiliation, receive aggression) and [2] the characteristics of the social partner, such as an adult male, subadult female, or mother carrying an infant (see Additional file 4: Table S5 for classification details). In contexts where the caller was either giving or receiving a social interaction, we only retained the ones for which the contextual features corresponding to social interaction partner attributes (i.e. sex and age class) could be identified (see Additional file 4: Tables S5 and S6 for details).

For social interactions involving a mother carrying an infant, we recorded the distance between the mother and the infant, categorising it as ‘carried on the belly’, ‘close’ (less than one metre), or ‘far’ (more than 1 m). This distinction was important because female sooty mangabeys frequently vocalise while in social contexts with infants of other females [70]. When the infant was more than 1 m away from the mother, we treated the infant as an independent social partner (IN). When the infant was carried or close to the mother, we treated the mother and the infant as a unit (MI) unless the behaviour received was affiliative, in which case we could identify whether the interaction was specifically directed at the mother (XM), the infant (XI) or both (XX). This level of detail could only be applied to affiliative interactions as the receiver could be reliably determined. For faster-paced interactions, specifically during aggression or approaches, the specific target (i.e. mother, infant, or both) could not always be reliably determined and hence the receiver was not assigned for these interactions. We also created a specific contextual feature for instances in which the caller directed gaze exclusively at the infant (IZ), without any accompanying behaviour. This applied regardless of whether the infant was close (< 1 m) or far (> 1 m) away from the mother.

When vocalisations occurred outside of social interactions, we assessed whether they were produced during environmental changes, such as encounters with other animal species, intergroup encounters with conspecifics, or after hearing alarm calls from other monkey species, or during other specific contexts that could elicit vocal production, such as the arrival of an adult male in the vicinity.

If none of these above features applied (which occurred in 53% of vocal utterances (*N* = 928) with clearly identifiable context), we assigned the activity of the caller as the contextual feature. As sooty mangabeys typically paused their activity to vocalise, making it difficult to assess what they were doing at the exact moment, we defined the activity based on the activity the caller was engaged in immediately before and after vocal production. If the caller engaged in the same activity before and after vocalisation, we categorised it as a stable activity (e.g. feeding, resting, moving). If the activity changed after vocalisation, we categorised it as a transitional activity (e.g. feeding, vocalising, then resting).

We conducted four to five separate inter-observer reliability tests with each data collector on focal behavioural data collection with Kappa scores ranging from 0.72 to 1 to assess the consistency of contextual feature classification (see Additional file 4: Table S7 for details).

In total, our continuous focal observations on adult sooty mangabeys resulted in the following data: TCP females, *N* = 17 individuals, *N* = 232 h 27 min (mean per individual = 13 h 40 min, SE = 0 h 56 min); TMP females, *N* = 18 individuals, *N* = 262 h 45 min (mean = 14 h 35 min, SE = 3 h 28 min); TCP males, *N* = 8 individuals, *N* = 122 h 21 min (mean = 15 h 17 min, SE = 1 h 39 min); TMP males, *N* = 4 individuals, *N* = 10 h 30 min (mean = 2 h 37 min, SE = 0 h 24 min). In addition, we recorded vocal utterances from ad libitum individuals (i.e., individuals who were not focal subjects): *N* = 4 TCP females, *N* = 2 TMP females, and *N* = 5 TMP males.

#### Audio recording coding

ALF, NB and TSA analysed the audio recordings using Raven Pro (v1.6.5 and 1.6.4) [96]. We manually inspected spectrograms (FFT size 1024, Hann window, hop size 256 samples) while simultaneously listening to the recordings to identify and annotate individual vocal elements (see glossary), marking their start and end times. We relied on the spoken vocal notes recorded in the field to identify the calls produced by the focal individuals. For classification, we referred to the established sooty mangabey vocal repertoire [70] and identified ten distinct call types: ‘grunt’, ‘twitter’, ‘growl’, ‘scream’, ‘grumble’, ‘vibrato’ (previously termed ‘copulation call’ in Range & Fischer’s repertoire), ‘whoop-gobble’, ‘wau’, ‘shrill’ (corresponding to the initial high-frequency elements of the alarm call as described in Range & Fischer’s repertoire) and ‘hoo’ (which includes both the ‘hoo’ call described by Range & Fischer and the final low-frequency sound element categorised initially as part of the alarm call). Additional details on each vocal element type, including spectrographic examples and definitions based on Range & Fischer’s repertoire, are provided in Additional file 5: Document S1.

To assess inter-rater reliability, ALF independently recoded *N* = 320 calls from NB sample (14% of NB sample) and *N* = 116 from TSA sample (21% of TSA sample), randomly selected. Agreement was high (Cohen’s kappa 0.96 for NB, 0.95 for TSA).

### Vocal utterances studied

We separated adult male and female vocal utterances, as they do not share the same vocal repertoire of single calls and therefore do not produce comparable sequences [65, 70].

The most frequent sequences produced by females were composed exclusively of ‘grunt’ and ‘twitter’ calls, occurring both as bigrams (‘grunt_twitter’ and ‘twitter_grunt’) and as longer iterative forms (e.g. ‘grunt_twitter_grunt’, ‘twitter_grunt_twitter_grunt’) (see Tables S5 and S6 for details). To analyse these sequences while accounting for iteration, we grouped sequences longer than two calls into broader categories: ‘grunt_twitterX’ and ‘twitter_gruntX’. These categories include iterations (i.e. non-adjacent repetitions of the same call types, see glossary), ranging from three to seven calls for ‘grunt_twitterX’ and from three to 29 calls for ‘twitter_gruntX’. This approach allowed us to examine structural features such as iteration and call order, while avoiding fine-grained categorisation based on the exact number of call reoccurrences, which varied across individuals (Table S5), thus limiting sample size. The final categories used in female analyses were: ‘grunt_twitter’, ‘grunt_twitterX’, ‘twitter_grunt’, and ‘twitter_gruntX’, along with all calls produced singly: ‘grunt’, ‘twitter’, ‘growl’, ‘scream’, ‘shrill’, and ‘vibrato’. Sequences composed of other call types were recorded infrequently and were excluded from quantitative analyses, though they are reported descriptively in the Results section.

For males, most utterances recorded for which we could identify the contextual feature of production consisted of single calls, and sequences involving multiple call types were infrequent and typically produced by only one to three individuals (Additional file 2: Table S4). The most common sequence was ‘shrill_hoo’. However, no single ‘shrill’ or ‘hoo’ calls were recorded in males, preventing testing the compositional nature of the ‘shrill_hoo’ sequence. Given this distribution, we did not test for contextual differences between single calls, bigrams and longer sequences in males. However, we report sample sizes and associated contexts for the main vocal utterances (i.e. single ‘grunt’ and ‘growl’ calls and ‘shrill_hoo’ bigram) in the Results section.

### Analyses

To investigate whether call combinations exhibit different contextual usage compared to their component calls (i.e. potential meaning shifts), we used the same analytical approach as a recent study on wild chimpanzees [33]. We used Euclidean distances that are commonly employed in experimental linguistics to assess phonological similarity between word pairs [97, 98]. In animal communication research, larger Euclidean distances indicate greater differences in contextual usage between utterances, suggesting potential meaning differences [33, 42].

We implemented Bayesian multinomial models in Stan via the ‘cmdstanr’ interface in R [99–101] to estimate the probability that each utterance type occurred in each of the 37 contextual features. The model included utterance type (10 levels: six single call types and four sequence types) and caller identity as varying intercepts and was run with four chains of 500 iterations each (plus 1000 iterations for warm-up), yielding 2000 posterior draws. We decided to include utterance type as a random intercept and not a fixed effect in the statistical models since the number of levels for this variable was large enough to estimate the value for each level as a random effect. Adding it as a fixed effect would drastically increase the complexity of the model without adding precision. From these posterior distributions of context probability per utterance type, we computed Euclidean distances between all pairs of utterance types. Because some utterances occurred in two contextual features simultaneously (i.e. social interaction + partner attributes; 35% of utterances), while others occurred in only one (e.g. feeding), we adjusted the probability vectors by the expected number of contextual features per utterance type before computing distances. This ensured that the Euclidean distance calculations accurately reflected the true contextual distributions. Full technical details, including the probability vector adjustment procedure and distance computation methods, are provided in Additional file 6: Document S2.

To formally test whether specific sequences composed of ‘grunt’ and ‘twitter’ calls were more strongly associated with the four contextual features most often associated with these vocal sequences (i.e. feed, give affiliation, receiver = infant and receiver = infant and mother), we compiled the posterior distribution of the proportion of association of each contextual feature with each utterance type from the multinomial model described above. This allowed us to get a measurement by pair of utterance types within context and better understand which utterance types sooty mangabeys tend to favour over others in a specific context.

We ensure sufficient sample size for this analysis that included the single calls ‘twitters’ (*N* = 119; 29 individuals) and ‘grunts’ *(N* = 890; 36 individuals) and the four types of vocal sequences comprising these calls (‘grunt_twitter’ (*N* = 76; 26 individuals), ‘twitter_grunt’ (*N* = 43; 17 individuals), ‘grunt_twitterX’ (*N* = 65; 18 individuals), ‘twitter_gruntX’ (*N* = 30; 11 individuals)), together with the contextual features: feed (*N* = 577 utterances, 36 individuals), give affiliation (*N* = 257 utterances, 27 individuals), receiver is an infant carried or close to their mother (*N* = 136 utterances, 25 individuals), and receivers are both infant and their nearby mothers (carried or close; *N* = 84, 21 individuals). We thus extracted four posterior distributions (one for each contextual feature) for each of the six utterance types (two single call types and four sequence types), resulting in 24 posteriors. To compare whether one utterance type occurred more often than another within the same contextual feature, we calculated the difference in the posterior samples of the proportion of that contextual feature for the two utterances. Specifically, for each posterior draw, we subtracted the posterior proportion of the contextual feature for utterance A from that for utterance B. We then determined the percentage of draws where this difference was positive, indicating how strongly the data supported utterance B being more frequent than utterance A in that contextual feature. This procedure generated a matrix of pairwise comparisons that reflected the differences in the strength of contextual feature association between each pair of utterances.

Note on adjacent repetitions: while adjacent repetitions (e.g. A_A_A) can alter sequence meaning [45, 102], our analysis focused on the type and order of calls within sequences. In this context, adjacent repetitions such as A_A_A are considered as three vocal elements forming one instance of the call type A (see glossary for definitions). This decision was motivated by the considerable variation in repetition length across call types [70], which would have required distinct modelling strategies beyond the scope of the present study.

## Supplementary Information

Additional file 1: Abstract in French

Additional file 2: Tables S1-S4-Sample sizes of vocal utterances

Additional file 3: Figure S1-Euclidean distances to ‘grunt’ and ‘twitter’

Additional file 4: Tables S5-S7-Context classification details

Additional file 5: Document S1-Vocal repertoire

Additional file 6: Document S2-Euclidean analysis details

## Data Availability

The data used in this study and the original code have been deposited at a Figshare repository and are publicly available at (10.6084/m9.figshare.29503334.v2) [103].
