# Supplementary material for: Call combination order and iterations may shift meaning in sooty mangabey vocal sequences
Source: BMC Biol. 2026 Feb 21;24:81. doi: 10.1186/s12915-026-02528-4 (PMC13032478; doi:10.1186/s12915-026-02528-4)
Supplement: Supplementary file 1 — Additional file 1: Abstract in French [file 12915_2026_2528_MOESM1_ESM.docx]

**Additional file 1**

**Abstract in French**

Publisher’s note:

This translation in French was submitted by the authors and we reproduce it as supplied. It has not been peer reviewed. Our editorial processes have only been applied to the original abstract in English, which should serve as reference for this article. This translated abstract is published under the same licence as the article.

**Résumé**

Contexte: Le langage humain se distingue par sa capacité à créer du sens grâce à des combinaisons de sons organisées selon des règles. Cette aptitude, longtemps considérée comme propre à notre espèce, est aujourd’hui observée chez certains animaux non humains, notamment chez les grands singes où des changements de sens ont été documentés lorsque des cris sont combinés en séquences. Chez les autres espèces, en revanche, les preuves de cette capacité restent limitées, et se concentrent principalement sur des contextes d’alerte. Pour explorer si cette faculté s’étend à d’autres contextes sociaux ainsi et à d’autres espèces, nous avons mené une analyse quantitative du répertoire vocal du mangabey fuligineux *(Cercocebus atys)*, un singe forestier d’Afrique de l’Ouest.

Résultats: Nous avons enregistré 1 751 vocalisations de deux groupes dans le parc national de Taï (Côte d’Ivoire). En utilisant le contexte de production comme indicateur du sens, nous avons examiné deux mécanismes : (1) Les bigrammes, des séquences de deux cris différents et (2) l’itération, la répétition d’un cri intercalé avec d’autres. Les analyses montrent que l’ordre des cris dans les bigrammes semble porteur de sens chez les femelles combinant les cris « grunt » et « twitter » : les cris isolés « grunt », « twitter », et le bigramme « grunt_twitter » apparaissent surtout lors de contextes de nourrissage. Le bigramme « twitter_grunt », en revanche, est principalement utilisé dans des interactions affiliatives, souvent dirigées vers les jeunes. Les séquences itératives comme « twitter_grunt_twitter », toujours produites par les femelles, montrent aussi des variations contextuelles, suggérant que l’itération pourrait moduler le sens.

Conclusions: Ces résultats indiquent que la capacité à générer du sens par combinaison vocale n’est pas limitée aux grands singes ni aux situations d’alerte : elle peut aussi s’appliquer à des interactions sociales positives chez les petits singes. Cela élargit notre compréhension de l’évolution des systèmes de communication. Toutefois, une utilisation étendue des bigrammes pour créer du sens, couvrant l’ensemble du répertoire vocal, n’a pas encore été démontrée chez les espèces non hominidés.

Mots-clés : compositionnalité, évolution du langage, séquences vocales, sens
