# Supplementary material for: Call combination order and iterations may shift meaning in sooty mangabey vocal sequences
Source: BMC Biol. 2026 Feb 21;24:81. doi: 10.1186/s12915-026-02528-4 (PMC13032478; doi:10.1186/s12915-026-02528-4)
Supplement: Supplementary file 2 — Additional file 2: Tables S1-S4-Sample sizes of vocal utterances [file 12915_2026_2528_MOESM2_ESM.docx]

**Additional file 2**

**Table S1.** **Adult female vocal utterances.**

The table below presents the recorded vocal utterance types, along with sample sizes of adult females for which we could define the context of production. ⁿ indicates that for each call, any number of repetitions of vocal elements is possible (from zero upwards).

| **Utterance type** | **N utterances (total = 1591)** | **N individuals (total = 41)** |
| --- | --- | --- |
| gruntⁿ | 890 | 36 |
| growlⁿ | 60 | 26 |
| gruntⁿ_twitterⁿ | 76 | 26 |
| twitterⁿ | 119 | 29 |
| screamⁿ | 43 | 20 |
| gruntⁿ_twitterⁿX | 65 | 18 |
| twitterⁿ_gruntⁿ | 43 | 17 |
| shrillⁿ | 26 | 15 |
| vibratoⁿ | 107 | 12 |
| twitterⁿ_gruntⁿX | 30 | 11 |
| growlⁿ_screamⁿ | 9 | 6 |
| screamⁿ_growlⁿ | 4 | 4 |
| shrillⁿ_hooⁿ | 9 | 3 |
| growlⁿ_grumbleⁿ_growlⁿ | 2 | 2 |
| growlⁿ_screamⁿ_growlⁿ | 2 | 2 |
| screamⁿ_growlⁿ_screamⁿ | 2 | 2 |
| grumbleⁿ_growlⁿ | 1 | 1 |
| growlⁿ_hooⁿ_growlⁿ_grumbleⁿ_growlⁿ | 1 | 1 |
| growlⁿ_hooⁿ_growlⁿ_hooⁿ_growlⁿ | 1 | 1 |
| growlⁿ_hooⁿ_growlⁿ_hooⁿ_growlⁿ_hooⁿ_growlⁿ | 1 | 1 |
| growlⁿ_hooⁿ_growlⁿ_hooⁿ_growlⁿ_hooⁿ_growlⁿ_hooⁿ_growlⁿ_hooⁿ_growlⁿ_hooⁿ_grumbleⁿ_hooⁿ_grumbleⁿ_growlⁿ_grumbleⁿ_hooⁿ_growlⁿ_hooⁿ_grumbleⁿ_growlⁿ_hooⁿ_growlⁿ_hooⁿ_grumbleⁿ_hooⁿ_growlⁿ_grumbleⁿ_growlⁿ_hooⁿ_growlⁿ_grumbleⁿ_growlⁿ_hooⁿ_growlⁿ_hooⁿ_growlⁿ | 1 | 1 |
| growlⁿ_screamⁿ_growlⁿ_grumbleⁿ | 1 | 1 |
| growlⁿ_screamⁿ_growlⁿ_screamⁿ_hooⁿ_growlⁿ_screamⁿ_hooⁿ | 1 | 1 |
| screamⁿ_hooⁿ | 1 | 1 |
| screamⁿ_hooⁿ_screamⁿ | 1 | 1 |
| screamⁿ_hooⁿ_screamⁿ_hooⁿ_screamⁿ | 1 | 1 |
| screamⁿ_twitterⁿ_screamⁿ_growlⁿ | 1 | 1 |
| shrillⁿ_hooⁿ_shrillⁿ | 1 | 1 |
| twitterⁿ_growlⁿ | 1 | 1 |
| twitterⁿ_growlⁿ_shrillⁿ_twitterⁿ_growlⁿ | 1 | 1 |

**Table S2. Adult female vocal sequences of grunts and twitters sampled from audio recordings.**

The table below presents the raw sequences, composed of ‘grunt’ and ‘twitter’ calls, with sample sizes of adult females for which we could define the context of production. ⁿ indicates that for each call, any number of repetitions of vocal elements is possible (from zero upwards).

| **Utterance type** | **N utterances** | **N individuals** |
| --- | --- | --- |
| gruntⁿ_twitterⁿ | 76 | 26 |
| gruntⁿ_twitterⁿ_gruntⁿ | 38 | 14 |
| gruntⁿ_twitterⁿ_gruntⁿ_twitterⁿ | 3 | 2 |
| gruntⁿ_twitterⁿ_gruntⁿ_twitterⁿ_gruntⁿ | 15 | 7 |
| gruntⁿ_twitterⁿ_gruntⁿ_twitterⁿ_gruntⁿ_twitterⁿ | 2 | 2 |
| gruntⁿ_twitterⁿ_gruntⁿ_twitterⁿ_gruntⁿ_twitterⁿ_gruntⁿ | 5 | 3 |
| gruntⁿ_twitterⁿ_gruntⁿ_twitterⁿ_gruntⁿ_twitterⁿ_gruntⁿ_twitterⁿ_gruntⁿ | 2 | 2 |
| twitterⁿ_gruntⁿ | 43 | 17 |
| twitterⁿ_gruntⁿ_twitterⁿ | 8 | 4 |
| twitterⁿ_gruntⁿ_twitterⁿ_gruntⁿ | 9 | 6 |
| twitterⁿ_gruntⁿ_twitterⁿ_gruntⁿ_twitterⁿ | 2 | 2 |
| twitterⁿ_gruntⁿ_twitterⁿ_gruntⁿ_twitterⁿ_gruntⁿ | 2 | 2 |
| twitterⁿ_gruntⁿ_twitterⁿ_gruntⁿ_twitterⁿ_gruntⁿ_twitterⁿ | 1 | 1 |
| twitterⁿ_gruntⁿ_twitterⁿ_gruntⁿ_twitterⁿ_gruntⁿ_twitterⁿ_gruntⁿ | 4 | 4 |
| twitterⁿ_gruntⁿ_twitterⁿ_gruntⁿ_twitterⁿ_gruntⁿ_twitterⁿ_gruntⁿ_twitterⁿ | 1 | 1 |
| twitterⁿ_gruntⁿ_twitterⁿ_gruntⁿ_twitterⁿ_gruntⁿ_twitterⁿ_gruntⁿ_twitterⁿ_gruntⁿ | 1 | 1 |
| twitterⁿ_gruntⁿ_twitterⁿ_gruntⁿ_twitterⁿ_gruntⁿ_twitterⁿ_gruntⁿ_twitterⁿ_gruntⁿ_twitterⁿ_gruntⁿ_twitterⁿ_gruntⁿ_twitterⁿ_gruntⁿ | 1 | 1 |
| twitterⁿ_gruntⁿ_twitterⁿ_gruntⁿ_twitterⁿ_gruntⁿ_twitterⁿ_gruntⁿ_twitterⁿ_gruntⁿ_twitterⁿ_gruntⁿ_twitterⁿ_gruntⁿ_twitterⁿ_gruntⁿ_twitterⁿ_gruntⁿ_twitterⁿ_gruntⁿ_twitterⁿ_gruntⁿ_twitterⁿ_gruntⁿ_twitterⁿ_gruntⁿ_twitterⁿ_gruntⁿ_twitterⁿ_gruntⁿ_twitterⁿ | 1 | 1 |
