# Supplementary material for: Call combination order and iterations may shift meaning in sooty mangabey vocal sequences
Source: BMC Biol. 2026 Feb 21;24:81. doi: 10.1186/s12915-026-02528-4 (PMC13032478; doi:10.1186/s12915-026-02528-4)
Supplement: Supplementary file 3 — Additional file 3: Figure S1-Euclidean distances to ‘grunt’ and ‘twitter’ [file 12915_2026_2528_MOESM3_ESM.docx]

**Additional file**
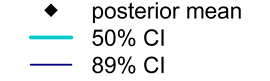
**3**


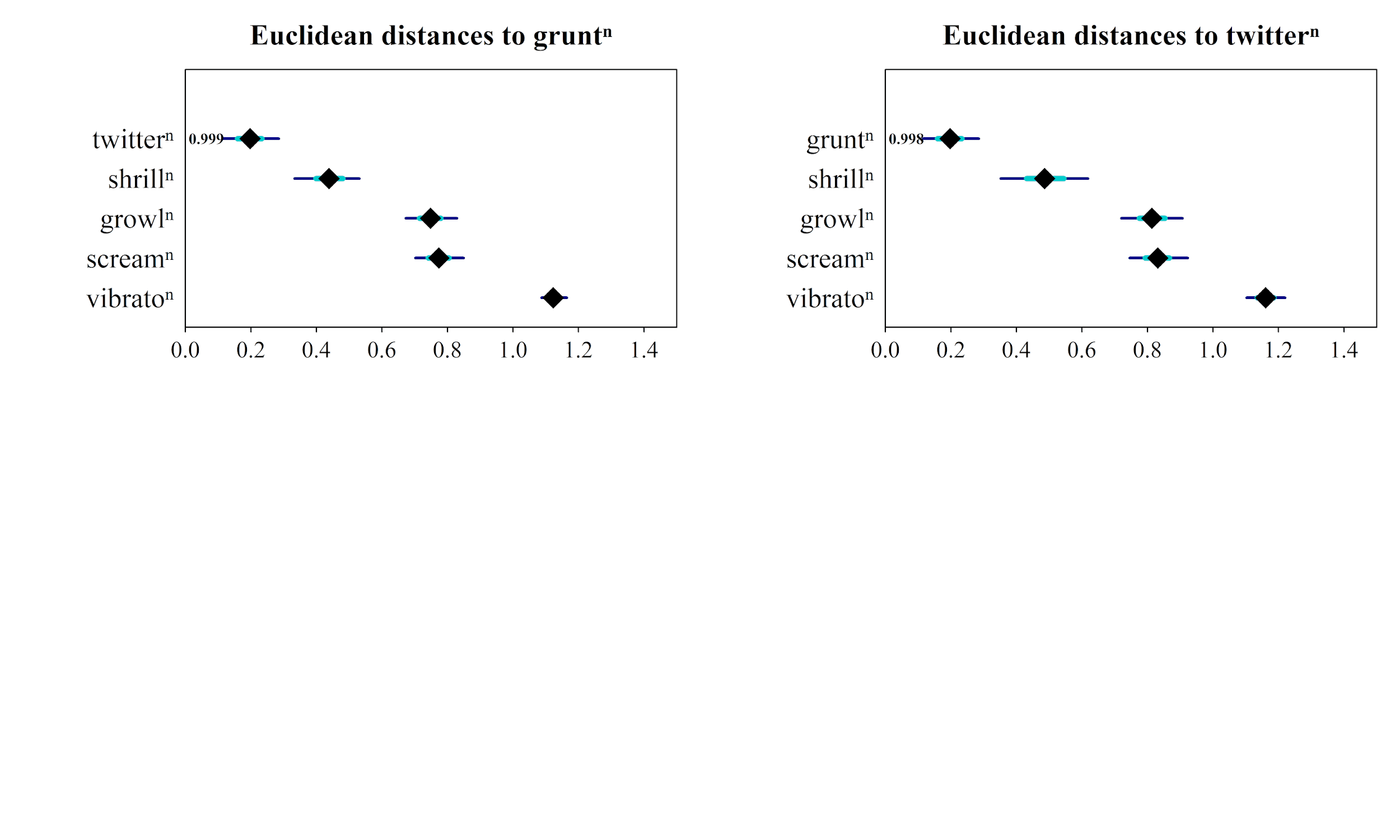


**Figure S1. Euclidean distances used to measure differences in contextual features distribution between ‘grunt’ and ‘twitter’ to other call types produced in isolation.** For each call, the mean distance and the 50% and 89% credible intervals relative to each comparison call are shown. Values on the Y-axis indicate the proportion of posterior samples in which the corresponding call was identified as the closest match to the focal call; only values greater than 0.01 are displayed. Superscript *n* indicates that each call can have any number of vocal element repetitions (from zero upwards).
