# Supplementary material for: Call combination order and iterations may shift meaning in sooty mangabey vocal sequences
Source: BMC Biol. 2026 Feb 21;24:81. doi: 10.1186/s12915-026-02528-4 (PMC13032478; doi:10.1186/s12915-026-02528-4)
Supplement: Supplementary file 4 — Additional file 4: Tables S5-S7-Context classification details [file 12915_2026_2528_MOESM4_ESM.docx]

**Additional file 4**

**Table S5.** **Ethogram of contextual features surrounding vocal** **production.** Based on the ethogram from Range & Fischer (2002), this table lists all features and their associated definitions. Contextual features frequently associated with vocal production in the literature are marked with an asterisk (*); see references below the table. The caller’s role (actor or receiver) is indicated for each type of social interaction, except in cases where the caller was a bystander to aggression (four observations), involved in a supplant (three observations), or engaged in copulation (which always occurred between a mature male and female). Whenever the caller’s role is specified, the interaction partner is considered part of the context.

| **Contextual feature type** | **Code** | **Definition** |
| --- | --- | --- |
| Stable activity | FE* | Feeding. Can be either:  -Eating: individual sits on the ground or in a tree and puts food in their mouth continuously.  -Foraging: individual moves slowly forward while visually scanning the forest floor and the lower parts of vegetation, occasionally putting food in their mouth. |
|  | MO* | Moving: individual walks steadily forward without visually scanning the forest floor and the lower parts of vegetation in search of food. |
|  | RE | Resting: individual rests or sleeps while sitting or lying down and can occasionally empty their cheek pouches and chew some food. |
| Transitional activity | FM* | Transition from feeding to moving. |
|  | FR | Transition from feeding to resting. |
|  | MF | Transition from moving to feeding. |
|  | MR | Transition from moving to resting. |
|  | RF | Transition from resting to feeding. |
|  | RM* | Transition from resting to moving. |
| Social interaction | GM/MG* | Give grooming/Receive grooming.  Continuous grooming interaction between individuals, excluding other affiliative behaviours. Recorded as a bout. |
|  | GA/RA* | Give approach/receive approach.  Approach: one individual moves towards another (the receiver), closing the distance until they are within one meter of each other, and positions their body towards the receiver. |
|  | GG/RG/BG* | Give aggression/receive aggression/bystander of an aggression.  Aggression: comprises hard contacts (i.e., biting, gripping, slapping), arm wave, chase (i.e., running after another individual), charge (i.e., changing speed while coming at another one while staring at them to displace them), display (stares at another individual while showing their teeth/tongue and/or head bobbing). |
|  | GI/RI* | Give affiliation/receive affiliation.  Affiliation: comprises embrace (i.e., one individual puts one or two arms around another individual), infant handling/touching (when the infant is carried by the mother or less than one meter from her), start grooming (i.e., first grooming contact initiated toward another individual), genital inspection, hugging using the tail (one individual wraps their tail around another one), mouth sniffing, mounting (i.e., copulation-like interaction but without penetration), play, genital presenting (i.e., one individual shows their genitals to another one by placing their body so the genitals are in front of the other’s face). |
|  | GZ | Gaze: one individual standing or sitting still and looking in the direction of another individual without engaging in any other social behaviour.    In sooty mangabeys, gaze is difficult to assess in the wild due to frequent and rapid head movements. Nevertheless, we systematically assessed gaze whenever a vocalisation was recorded. We only included conspicuous gazes—those where the individual’s head remained still or moved slowly to follow the gaze target, and where the recipient or object of the gaze could be clearly identified within approximately 5 metres. If gaze occurred alongside other social behaviours, we classified the context based on those behaviours, as gaze in such cases was considered part of a broader interaction rather than a distinct behavioural event. These strict criteria likely contributed to the limited number of gaze observations in our dataset. |
|  | SU | Supplant: an individual (the actor) approaches another individual (the receiver) occupying a resource site (i.e., a 1-metre circumference area containing food around the receiver) without displaying aggression. The receiver is either sitting and eating or standing and foraging. The receiver immediately leaves the site upon the actor’s approach, either at a normal speed, quickly, or even fleeing. The actor then takes the receiver's place at the site to eat or forage. |
| Environmental changes | AE* | Animal encounter: encounter with another animal (i.e., eagle, chimpanzee, leopard, Gabon/rhinoceros viper, hippo, duiker, squirrel). |
|  | IT | Intergroup encounter: encounter with a neighbouring group of sooty mangabeys. |
|  | MK* | Monkey alarm: alarm call given by another monkey species (i.e., black-and-white colobus, Campbell’s monkey, Diana monkey, lesser spot-nosed monkey). |
|  | TR | Tree: falling branch or tree. |
| Other interactions | CO* | Copulation. For copulation, we did not assign a partner as contextual feature , as these interactions always involved a mature male and a mature female, and we had no rationale to expect differences in vocal production based on the age class (subadult vs. adult). |
|  | MA | Male adult arrival: arrival of an adult male from the group at the location of the caller. |

References:

Grampp, M., Samuni, L., Girard-Buttoz, C., León, J., Zuberbühler, K., Tkaczynski, P., ... & Crockford, C. (2023). Social uncertainty promotes signal complexity during approaches in wild chimpanzees (Pan troglodytes verus) and mangabeys (Cercocebus atys atys). *Royal Society Open Science*, *10*(11), 231073.

León, J., Thiriau, C., Crockford, C., & Zuberbühler, K. (2023). Comprehension of own and other species’ alarm calls in sooty mangabey vocal development. *Behavioral Ecology and Sociobiology*, *77*(5), 56.

León, J., Quintero, F., & Zuberbühler, K. (2023). Acquisition of predator knowledge in sooty mangabeys. *Animal Behaviour*, *205*, 1-14.

Mielke, A., Crockford, C., & Wittig, R. M. (2019). Snake alarm calls as a public good in sooty mangabeys. *Animal Behaviour*, *158*, 201-209.

Neumann, C., & Zuberbühler, K. (2016). Vocal correlates of individual sooty mangabey travel speed and direction. *PeerJ*, *4*, e2298.

Quintero, F., Touitou, S., Magris, M., & Zuberbühler, K. (2022). The evolution of food calls: vocal behaviour of sooty mangabeys in the presence of food. *Frontiers in Psychology*, *13*, 897318.

Quintero, F., Touitou, S., Magris, M., & Zuberbühler, K. (2023). Audience Effects in Sooty Mangabey Agonistic Behaviour.

Range, F., & Fischer, J. (2004). Vocal repertoire of sooty mangabeys (Cercocebus torquatus atys) in the Taï National Park. *Ethology*, *110*(4), 301-321.

**Table S6.** **Social interaction partner with age-class classification.**

| Age Class | Code | Age/biological marker |
| --- | --- | --- |
| Infant | IN | <1 years old, more than 1m from the mother; for affiliation and aggression  Biological marker: mostly carried by mother, or close to mother. |
| Infant | IZ | <1 years old, more than 1m from the mother; as being the receiver of a gaze. |
| Juvenile | JU | Between >= 1 and < 4 years old for females  Between >= 1 and < 4 years old for males  Biological marker: not carried by mother, independent from mother. |
| Subadult female | SF | Between >= 4 and < 5 years old  Biological marker: first appereance of sexual swelling. |
| Subadult male | SM | Biological marker:  -min the size of an adult female.  -tests begin to appear and/or canines begin to develop. |
| Adult female not carrying an infant | AF | > 5 years old  Biological marker:  -full size.  -first parturition. |
| Adult female carrying/close to her infant (i.e., <1m) at the time of data collection for aggression and approaches (not affiliations) | MI |  |
| Infant being carried/being closed to (<1m) by/to their mother and being the receiver of an affiliation | XI |  |
| Adult female carrying/close to her infant (i.e., <1m) at the time of data collection being the receiver of an affiliation (not the infant) | XM |  |
| Adult female carrying/close to her infant (i.e., <1m) at the time of data collection and both her and her infant are being the receivers of an affiliation (i.e., behaviours of the actor are given to both) | XX |  |
| Adult male | AM | Biological marker:  -tests completely out and canines fully developed. |

**Table S7.** **Inter-observer tests on context classification.**

We conducted two sets of inter-observer reliability tests on context classification: one during the 2022 field session between ALF and TA, and another during the 2023 field session between ALF and NB. We used an unweighted Cohen’s kappa test and considered a value of at least 0.7 (i.e., substantial agreement) as satisfactory.

Gaze direction was recorded infrequently due to our strict and conservative criteria for assessing it (see Table S1, 'Gaze' in the ethogram).

We did not conduct a reliability test for mother–infant distance between ALF and TSA because data collection began in June, after infants were no longer being carried or staying close to their mothers.

Grooming was included under social interaction behaviours in the reliability test because it was initially not classified as an activity. We reclassified it post hoc to unify all long-duration behaviours and to distinguish them from brief affiliative events.

| **Category** | **ALF vs TSA** | **ALF vs NB** |
| --- | --- | --- |
| Activities | κ = 0.72, *z* = 6.17, *p* < 0.001, *N* = 50 observations | κ = 0.87, *z* = 7, *p* < 0.001, *N* = 64 observations |
| Social interaction behaviours | κ = 0.81, *z* = 12.4, *p* < 0.001, *N* = 23 observations | κ = 0.74, *z* = 20.1, *p* < 0.001, *N* = 63 observations |
| Caller role during social interaction (actor/receiver) | κ = 1, *z* = 7.88, *p* < 0.001, *N* = 18 observations | κ = 1, *z* = 6.08, *p* < 0.001, *N* = 37 observations |
| Gaze direction | κ = 1, *z* = 2.65, *p* < 0.01, *N* = 7 observations | κ = 0.73, *z* = 2.62, *p* < 0.01, *N* = 8 observations |
| Distance between a mother and their infant | - | κ = 0.81, *z* = 5.02, *p* < 0.001, *N* = 38 observations |
