# Supplementary material for: Call combination order and iterations may shift meaning in sooty mangabey vocal sequences
Source: BMC Biol. 2026 Feb 21;24:81. doi: 10.1186/s12915-026-02528-4 (PMC13032478; doi:10.1186/s12915-026-02528-4)
Supplement: Supplementary file 5 — Additional file 5: Document S1-Vocal repertoire [file 12915_2026_2528_MOESM5_ESM.docx]

**Additional file 5**

**Document S1.** **Adult sooty mangabey call types.**

Range and Fischer (2004) have thoroughly described the complete vocal repertoire of sooty mangabeys, and readers seeking more detail should refer to their paper. For convenience, we provide a condensed version here, including all the call types examined in our study. All images except from the “Wau” call originate from Sigmundson et. al (2025) with permission to use (https://creativecommons.org/licenses/by/4.0/).

*Growl*

Growls are characterised by a low fundamental frequency band with many visible overtones, and they vary in both frequency and duration, often comprising a combination of shorter and prolonged elements.


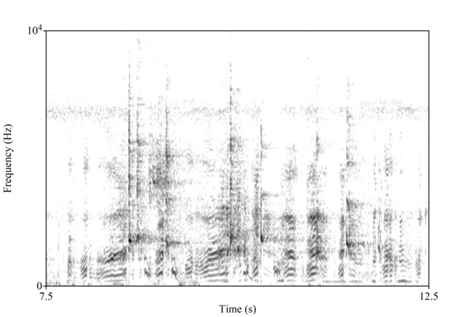


Spectrographic examples of growls. y axis represents the frequency in Hz and x axis the time in s.

*Grumble*

Grumbles are longer than growls, characterised by a low fundamental frequency and a rich harmonic structure with up to 12 overtones.


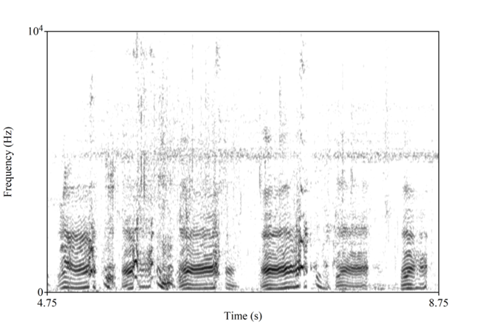


Spectrographic examples of grumbles interspersed with growls (shorter vocalisations). y axis represents the frequency in Hz and x axis the time in s.

*Grunt*

Grunts are low-frequency vocalisations, consistently short in duration (less than 200 milliseconds), and produced across a wide range of contexts. Adult females and males diverge in grunt production (see Range and Fischer).


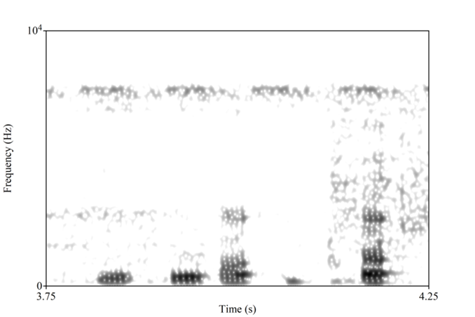

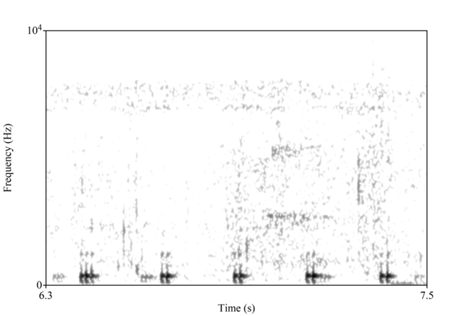


Spectrograms of grunt calls, from an adult female (left) and an adult male (right). The y axis represents the frequency in Hz and x axis the time in s.

*Scream*

Screams are harsh calls characterised by wide-band noise extending up to 15 kHz, often lasting for several seconds.


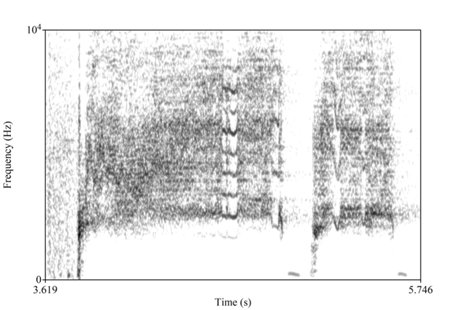

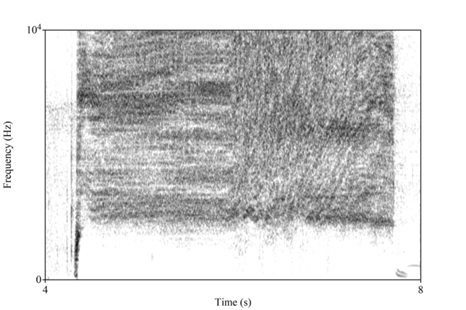


Spectrographic examples of screams. The y axis represents the frequency in Hz and the x axis is time in seconds (s).

*Shrill and hoo*

Referred to as the alarm call in Range and Fischer, 2004). Shrill calls consist of several high-frequency elements, sometimes displaying a harmonic structure, though this is less common, and are frequently combined with a hoo call, which features a low fundamental frequency and a rich, readily visible harmonic structure.


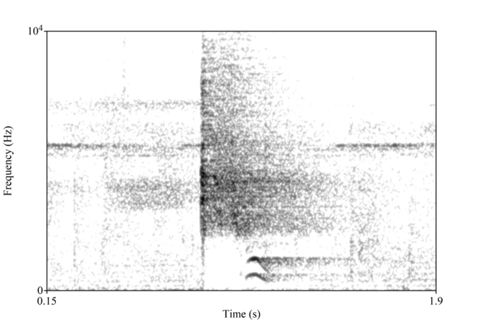

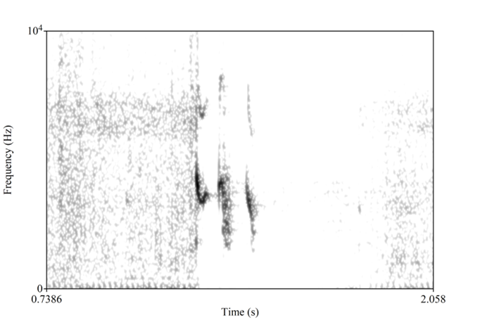


Spectrograms of shrill calls combined with a hoo call (left) and shrill call produced singly (right). The y axis represents the frequency in Hz and the x axis is time in s.

*Twitter*

Twitters are short, high-frequency calls with a fundamental frequency around 1 kHz and many visible overtones extending up to 20 kHz. Adult males do not produce them.


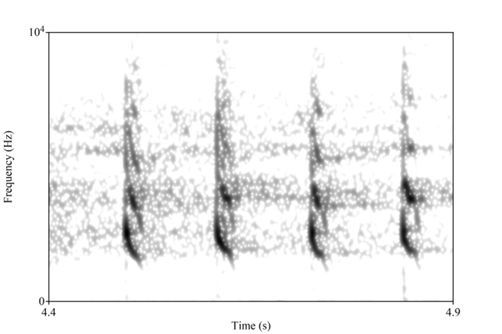


Spectrographic example of twitter. The y axis represents the frequency in Hz and the x axis is time in s.

*Vibrato*

Referred to as the copulation call in Range and Fischer, 2004. Complex call used exclusively by sexually mature females. It consists of many variable elements with a low fundamental frequency and a rich harmonic structure, with individual elements alternating between pitch shifts upwards and downwards, creating the characteristic "vibrato."


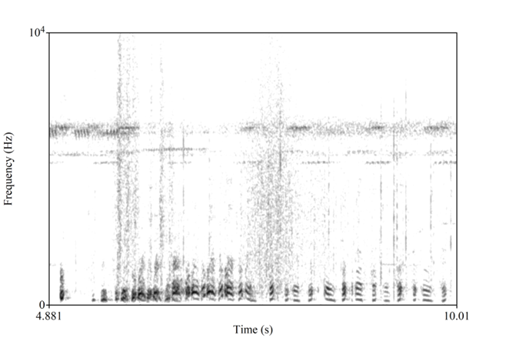


Spectrographic example of vibrato. The y axis represents the frequency in Hz and the x axis is time in s.

*Wau*

Short, low-frequency call with an ascending-descending pitch pattern and several visible harmonics.


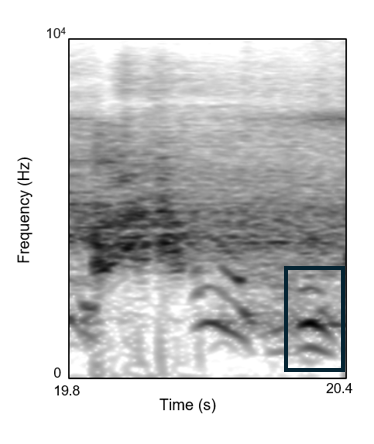


Spectrographic example of wau (in the black box) preceded by shrill and hoo. The y axis represents the frequency in Hz and the x axis is time in s.

*Whoop-gobble*

Loud, low-frequency complex call that can be heard over long distances and produced by adult males. It begins with a single low-frequency element, followed by a pause that may last several seconds, before concluding with a series of repetitive, low-frequency elements. Although the pause between the first and final elements typically violates our usual rule for defining a call type, we treated it as a special case due to its consistent production in this form.


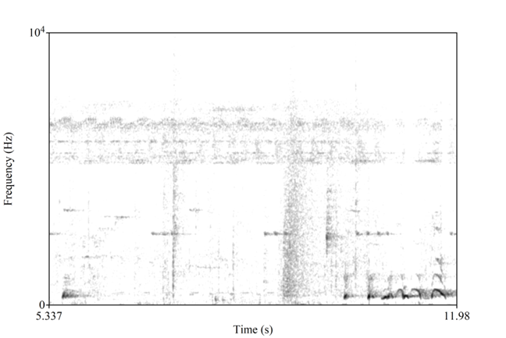


Spectrographic example of whoop-gobble. The y axis represents the frequency in Hz and the x axis is time in s.

Reference

Range, F., & Fischer, J. (2004). Vocal repertoire of sooty mangabeys (Cercocebus torquatus atys) in the Taï National Park. *Ethology*, 110(4), 301-321.

Sigmundson, R., Girard-Buttoz, C., Le Floch, A., Azaiez, T. S., McElreath, R., Zuberbühler, K., Wittig, R.M. & Crockford, C. (2025). Vocal sequence diversity and length remain stable across ontogeny in a catarrhine monkey (Cercocebus atys). *Communications Biology*, 8(1), 465.
