# Supplementary material for: Call combination order and iterations may shift meaning in sooty mangabey vocal sequences
Source: BMC Biol. 2026 Feb 21;24:81. doi: 10.1186/s12915-026-02528-4 (PMC13032478; doi:10.1186/s12915-026-02528-4)
Supplement: Supplementary file 6 — Additional file 6: Document S2-Euclidean analysis details [file 12915_2026_2528_MOESM6_ESM.docx]

**Additional file 6**

**Document S2. Euclidean analysis details**

Following Girard-Buttoz et al. 2025 we implemented a multinomial model in Stan via the ‘cmdstanr’ interface in R (99–101). The input data consisted of a binary matrix with 1459 rows (utterances) and 37 columns (contextual features), where each cell indicated the presence (1) or absence (0) of each contextual feature during a given utterance; for utterances involving two contextual features, both were included. Utterance type (a factor with 10 levels: six single call types and four sequence types, i.e., ‘twitter_grunt’, ‘grunt_twitter’, ‘twitter_gruntX’, ‘grunt_twitterX’) and caller identity were included as varying intercepts. The model was run with four chains, each with 500 iterations (and 1000 warm-up), yielding 2000 posterior draws.

To compute Euclidean distances between utterance types, we extracted from the multinomial model the posterior distribution of the probability vector of each contextual feature to occur for each utterance type, averaged across individuals (i.e., omitting the individual-level intercept). The estimated probabilities across the 37 contextual features for a given vocal utterance sum to 1. 35% of utterances (517 out of 1,549) occurred in two contextual features (i.e., social interaction + social partner attributes). To get a contextual feature distribution that mirrors this pattern, we multiplied each probability vector by the expected number of contextual features per recording of a given utterance type. For example, suppose utterance A was recorded three times: twice during give affiliation + adult female, and once during give approach + juvenile male. This yields raw contextual feature counts of 2 (affiliation), 2 (adult female), 1 (approach), and 1 (juvenile male), summing to 6 contextual features across 3 utterances, or 2.0 contextual features per occurrence. The corresponding raw probability vector is [2, 2, 1, 1]/6 = [0.33, 0.33, 0.17, 0.17]. This vector is then multiplied by the expected number of contextual features per utterance (2.0), yielding [0.66, 0.66, 0.33, 0.33] as the adjusted contextual feature distribution for utterance A. These adjusted vectors served as the basis for calculating the Euclidean distances between utterance types (see also (33)).

As the number of contextual features could vary depending on the utterance type, we modelled the “contextual feature length” (i.e., the number of contextual features associated with each utterance, one or two) using a zero-truncated Poisson likelihood, since each utterance had at least one associated contextual feature. Varying intercepts were included for utterance type and caller ID. From the posterior estimates of the model, we simulated 1,000 recordings per utterance type (where the contextual feature length was a random number drawn from a truncated Poisson distribution with the estimated mean for the current utterance type) and extracted which contextual feature(s) occurred. For each simulated recording, we computed the contextual feature-adjusted probability vector for the corresponding utterance type following the procedure outlined above. The outcome of these 1,000 simulations was then returned as a vector of means per contextual feature type. We repeated this procedure for all 10 utterance types, yielding a corresponding set of adjusted probability vectors. These vectors were then used to compute Euclidean distances between each pair of utterance types.
